# Proximity-based proteomics reveals the thylakoid lumen proteome in the cyanobacterium *Synechococcus* sp. PCC 7002

**DOI:** 10.1007/s11120-020-00806-y

**Published:** 2020-12-06

**Authors:** Kelsey K. Dahlgren, Colin Gates, Thomas Lee, Jeffrey C. Cameron

**Affiliations:** 1 Department of Biochemistry, University of Colorado, Boulder, CO 80309 USA; 2 Renewable and Sustainable Energy Institute, University of Colorado, Boulder, CO 80309 USA; 3 BioFrontiers Institute, University of Colorado, Boulder, CO 80309 USA; 4 Interdisciplinary Quantitative Biology Program (IQ Biology), BioFrontiers Institute, University of Colorado, Boulder, CO 80309 USA; 5 National Renewable Energy Laboratory, Golden, CO 80401 USA

**Keywords:** APEX2, Proximity-based proteomics, Thylakoid lumen, Cyanobacteria, Photosynthesis, Photosystem II

## Abstract

Cyanobacteria possess unique intracellular organization. Many proteomic studies have examined different features of cyanobacteria to learn about the intracellular structures and their respective functions. While these studies have made great progress in understanding cyanobacterial physiology, the conventional fractionation methods used to purify cellular structures have limitations; specifically, certain regions of cells cannot be purified with existing fractionation methods. Proximity-based proteomics techniques were developed to overcome the limitations of biochemical fractionation for proteomics. Proximity-based proteomics relies on spatiotemporal protein labeling followed by mass spectrometry of the labeled proteins to determine the proteome of the region of interest. We performed proximity-based proteomics in the cyanobacterium *Synechococcus* sp. PCC 7002 with the APEX2 enzyme, an engineered ascorbate peroxidase. We determined the proteome of the thylakoid lumen, a region of the cell that has remained challenging to study with existing methods, using a translational fusion between APEX2 and PsbU, a lumenal subunit of photosystem II. Our results demonstrate the power of APEX2 as a tool to study the cell biology of intracellular features and processes, including photosystem II assembly in cyanobacteria, with enhanced spatiotemporal resolution.

**Supplementary Information:**

The online version contains supplementary material available at 10.1007/s11120-020-00806-y.

## Introduction

The intracellular spatial organization of cyanobacteria is unique among prokaryotes. As Gram-negative bacteria, cyanobacteria possess the typical inner and outer membrane systems enclosing a cell wall comprised of peptidoglycan. However, most cyanobacterial species also possess thylakoid membranes, an extra set of intracellular membranes where photosynthesis occurs, as well as carboxysomes, proteinaceous organelles used for carbon fixation. The distinctive intracellular spatial organization and protein complexes found within cyanobacteria have drawn particular interest to the cell biology of these organisms. Furthermore, cyanobacteria can also be used as a model for plant chloroplasts, as they share structural and biochemical similarities and have a common evolutionary ancestor. As a result, many proteomic studies of specific cyanobacterial structures, i.e. thylakoid membranes, have been performed (Agarwal et al. 2010; Baers et al. 2019; Cheregi et al. 2015; Fulda et al. 2000; Gao et al. 2014a; Herranen et al. 2004; Huang et al. 2002, 2004, 2006; Kashino et al. 2002; Kurian et al. 2006a; Li et al. 2012; Liberton et al. 2016; Oliveira et al. 2016; Pisareva et al. 2007, 2011; Rajalahti et al. 2007; Rowland et al. 2010; Sergeyenko and Los 2000; Srivastava et al. 2005; Trautner and Vermaas 2013; Wang et al. 2000; Zhang et al. 2009). These studies have made great progress towards understanding the physiology of cyanobacteria, but lack the spatial resolution necessary to resolve the composition of many intracellular structures resistant to traditional biochemical fractionation and purification methodologies.

**Fig. 1:**
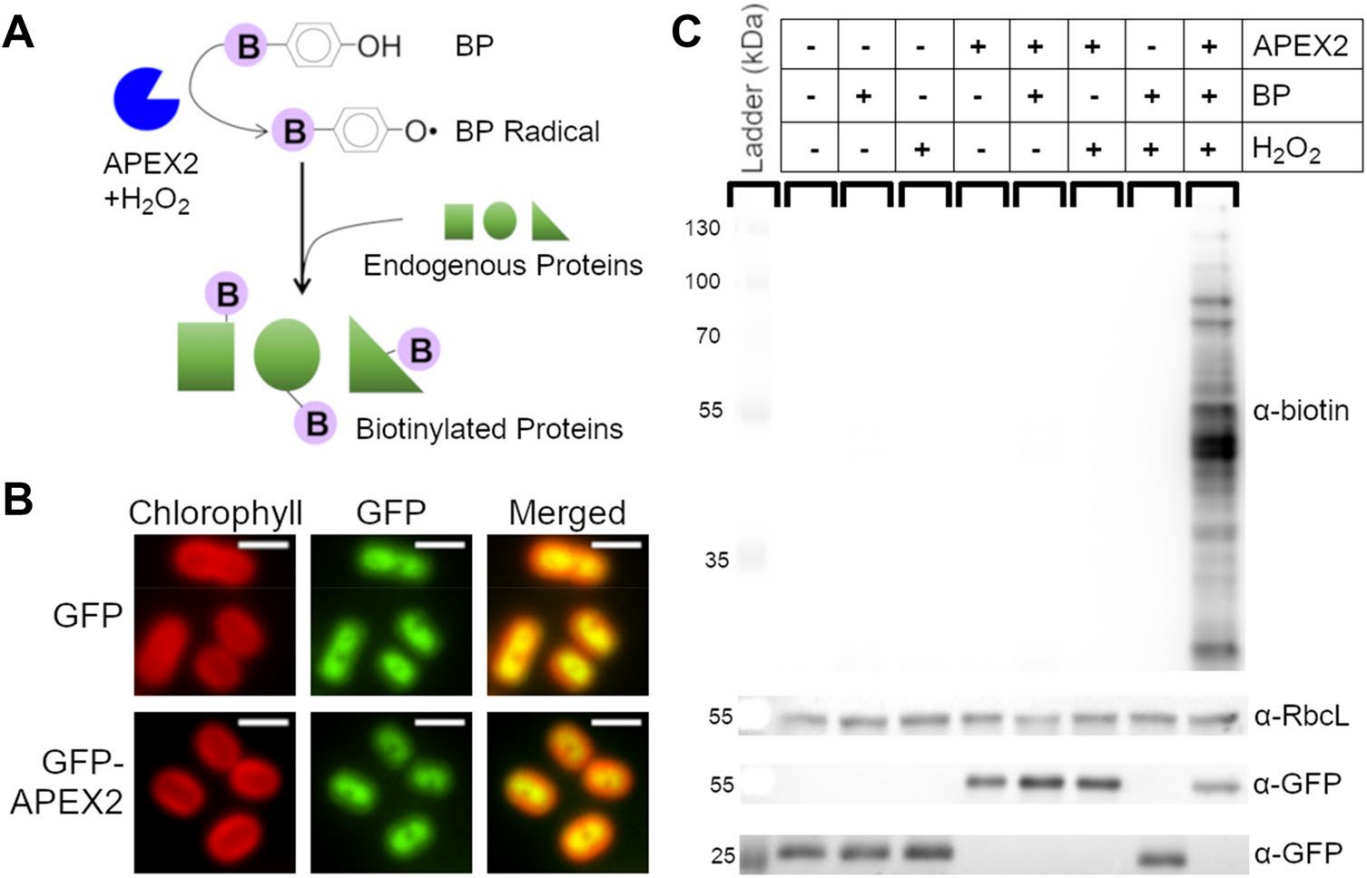
APEX2-dependent labeling specifically biotinylates proteins in PCC 7002. **a** APEX2 reacts with BP in the presence of H_2_O_2_ to produce a BP radical. Biotinylated proteins are generated when the BP radical reacts with peptides, forming a covalent bond. **b** Cells expressing GFP and GFP-APEX2 (green) imaged using fluorescence microscopy. Scale bars are 2 µm. Chlorophyll channel (red) indicates thylakoid membrane. **c** 5 µg of protein from cells expressing either GFP or GFP-APEX2 was separated by SDS-PAGE and transferred to a membrane for immunoblot analysis using streptavidin to detect APEX2 activity. anti-RbcL antibody was used as a loading control and the same membrane was stripped and re-probed with anti-GFP antibody to check for expression of GFP (28 kDa) or GFP-APEX2 (54 kDa)

Previously, proteomic studies of cyanobacterial components were limited to fractionation and separation techniques which could introduce artifacts and result in ambiguous cellular localizations. For example, mechanical disruption of cells often leads to cross-contamination between fractions and is, therefore, impractical for non-membrane-bound regions or complex structures such as the thylakoid lumen. However, a technique termed proximity-based proteomics was recently developed in mammalian cells to allow for proteomic analysis of cellular regions or protein interactomes that were unable to be purified using existing techniques (Kim and Roux 2016). Proximity-based proteomics relies on targeting a specific enzyme to a region of interest as a protein fusion to a full-length protein or signal sequence. The enzyme then performs chemistry in live cells to label proteins within a small radius (10–20 nm) of itself (Rhee et al. 2013). After cell lysis, the labeled proteins can then be separated from unlabeled proteins and analyzed using mass spectrometry. Several proximity-based proteomics techniques exist, but the most common use enzymes that biotinylate proteins (Kim and Roux 2016). We chose to use APEX2, an engineered ascorbate peroxidase that catalyzes a reaction between biotin-phenol (BP) and hydrogen peroxide (H_2_O_2_) to create a BP radical that covalently attaches to proteins (Hung et al. 2016; Lam et al. 2015) (Fig. 1a). The reactivity and short half-life of biotin-phenol gives this technique a high-spatial specificity. Furthermore, APEX2 has been shown to be catalytically active in multiple cellular compartments and exhibits a short (1 min) labeling time, allowing for high temporal specificity (Hung et al. 2016; Lam et al. 2015).

Here, we demonstrate the feasibility and potential of a proximity-based proteomics technique using APEX2 in *Synechococcus* sp. PCC 7002 (PCC 7002), a model cyanobacterium and promising chassis for biotechnological applications (Markley et al. 2015; Ruffing et al. 2016; Xu et al. 2011). To showcase the ability of APEX2 to interrogate regions of the cell where proteomics studies have not yet been possible due to limitations of existing biochemical methods, we targeted APEX2 to the thylakoid lumen by fusing it to PsbU, an extrinsic photosystem II (PSII) protein (Nishiyama et al. 1998), and identified the PsbU-associated proteome by mass spectrometry. Determining the thylakoid lumen proteome is vital for understanding the physiological roles of the thylakoid membrane system and the reactions of oxygenic photosynthesis.

## Results and discussion

### Characterization of APEX2 labeling in PCC 7002

To determine if APEX2-dependent labeling of proteins was possible in cyanobacteria, GFP or GFP-APEX2 was incorporated into the genome of PCC 7002. Cytoplasmic localization of GFP and GFP-APEX2 was confirmed using fluorescence microscopy (Fig. 1b). To perform APEX2-dependent biotinylation, cells were incubated with BP for 30 min and then exposed to H_2_O_2_ for 1 min. After quenching the reaction, cells were lysed by bead beating and a streptavidin blot confirmed the ability of APEX2 to biotinylate proteins in PCC 7002 (Fig. 1c). Biotin labeling was only detected in the presence of APEX2, BP, and H_2_O_2_, demonstrating reaction specificity in vivo. Furthermore, the rapid reaction enables precise temporal control of labeling.

### Purification of cytoplasmic APEX2-biotinylated proteins from PCC 7002

**Fig. 2:**
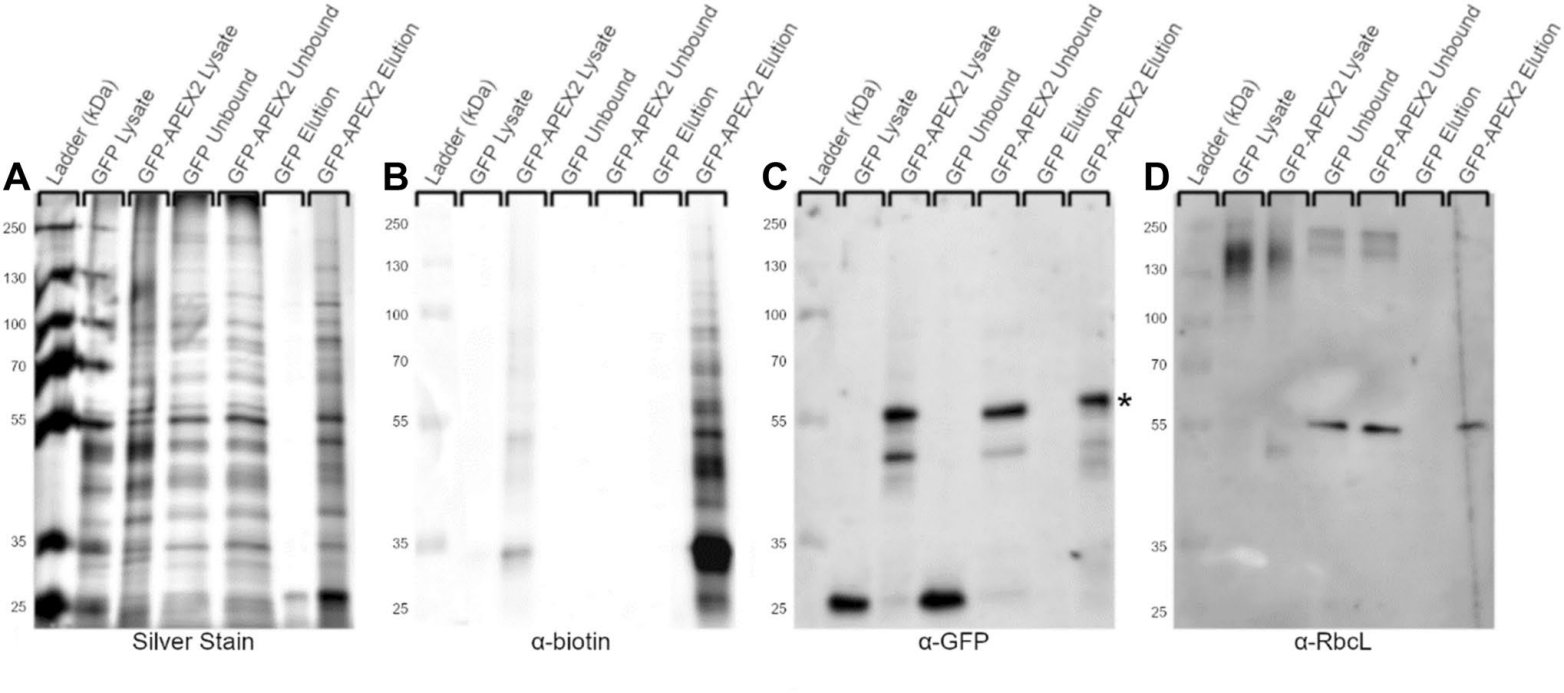
Enrichment of proteins biotinylated by cytoplasmic APEX2 in vivo. Cells expressing GFP or GFP-APEX2 were incubated with BP and exposed to H_2_O_2_. Biotinylated proteins were captured from cell lysates on streptavidin coated magnetic beads. Fractions from each enrichment step were separated by SDS-PAGE and then silver stained for contrast or transferred to a nitrocellulose membrane and probed with specific antibodies. **a** Silver stain of noted fractions from unlabeled (GFP) or labeled (GFP-APEX2) lysates. **b** Biotinylated proteins are only detected in fractions containing APEX2 and are enriched on streptavidin beads. **c** Expected self-labeling (biotinylation) of GFP-APEX2 (54 kDa, marked with *) is confirmed by immunoblotting against GFP. **d** RbcL (55 kDa), a cytoplasmic protein expected to be labeled by GFP-APEX2 was specifically captured on beads incubated with GFP-APEX2

Proteins biotinylated in vivo were enriched for further analysis by affinity purification. APEX2-dependent biotinylation was performed in cells expressing GFP or GFP-APEX2 in the cytoplasm. Affinity purification of biotinylated proteins was performed by incubating cellular lysates with streptavidin-coated magnetic beads. The background level of biotinylation was very low as biotinylated protein was only detected in cells expressing GFP-APEX2, but not cells expressing GFP alone (Fig. 2a, b). To confirm cytoplasmic APEX2 labels cytoplasmic proteins, immunoblots using antibodies against expected cytoplasmic proteins were performed (Fig. 2c, d). Since the BP radical reacts with proteins within a 10–20 nm radius of its origin, APEX2 itself is expected to be biotinylated. Biotinylated GFP-APEX2 fusion protein was detected using an anti-GFP antibody, confirming the expected self-reactivity (Fig. 2c). Additionally, the large subunit of rubisco (ribulose-1,5-bisphosphate carboxylase/oxygenase), RbcL, an abundant cytoplasmic protein, was only enriched on beads incubated with cells expressing GFP-APEX2 as detected using a specific anti-RbcL antibody (Fig. 2d). The high molecular weight RbcL band in lysates is likely the result of higher-order complexes formed in vivo; RbcL assembles into large protein assemblies to form the carboxysome, a bacterial microcompartment (Cameron et al. 2013). Following the more stringent enrichment and elution process, these complexes have been disrupted and RbcL migrates as expected.

### PsbU-APEX2 and cytoplasmic APEX2 label different sets of proteins

**Fig. 3:**
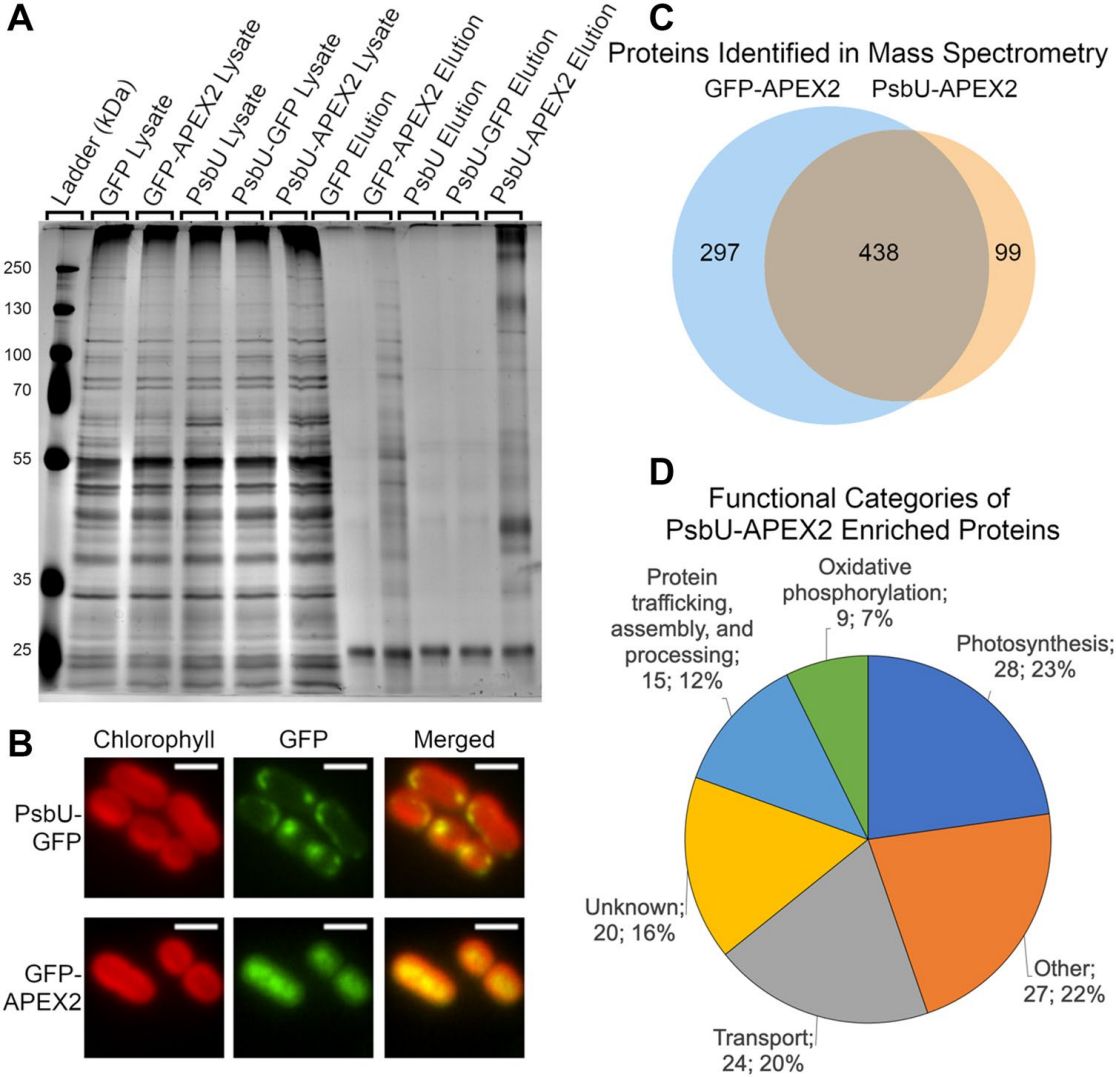
PsbU-APEX2 and Cytoplasmic APEX2 label different sets of proteins. **a** Silver stain of the biotinylated protein purification from PCC 7002 expressing GFP, GFP-APEX2, PsbU, PsbU-GFP, or PsbU-APEX2 after APEX2-dependent biotinylation. **b** Localization of PsbU-GFP and GFP-APEX2 were visualized with fluorescence microscopy (Green). Chlorophyll channel (red) indicates thylakoid membrane. Scale bars are 2 µm. **c** Biotinylated proteins from strains expressing GFP-APEX2 and PsbU-APEX2 identified by mass spectrometry. **d** Functional categories of the proteins enriched in PsbU-APEX2 samples obtained from quantitative analysis of mass spectrometry data (number of proteins; percentage of 123 total proteins). The proteins used for this analysis are listed in Table 1. (Also see Supplementary Tables 1 and 2)

APEX2 was fused to a protein localized to the thylakoid lumen to demonstrate the ability of proximity-based proteomics to interrogate subcellular regions that have not been successfully purified using traditional methods. To accomplish this, the localizations of several candidate proteins fused to GFP were examined by fluorescence microscopy. Of these candidates, PsbU, an extrinsic subunit of PSII, exhibited the most promising localization and therefore was selected to target APEX2 to the thylakoid lumen. The PsbU-APEX2 gene fusion is expressed from neutral site 1 in the chromosome under a constitutive promoter. APEX2-dependent labeling and biotinylated protein purification was performed on cells expressing thylakoid lumenal PsbU-APEX2 and cells expressing cytoplasmic GFP-APEX2. A silver stain of purified biotinylated proteins from GFP-APEX2 and PsbU-APEX2 shows different banding patterns, suggesting that a different set of proteins is labeled by the different APEX2 fusions (Fig. 3a). The thylakoid localization of PsbU-GFP was confirmed using fluorescence microscopy (Fig. 3b). The localization of PsbU-GFP was used as a proxy for the localization of PsbU-APEX2, since GFP and APEX2 are both C-terminal tags of a similar size. To identify the proteins labeled by the different APEX2 fusion proteins, biotinylated proteins were purified from two independent samples of both PsbU-APEX2 labeled and GFP-APEX2 labeled cells, and the resulting peptides following tryptic digestion were separated and detected using LC–MS/MS. Protein identification required a minimum of 2 spectral counts and 2 peptides in each sample. 99 proteins were identified exclusively in both PsbU-APEX2 replicates and 297 proteins were identified exclusively in both GFP-APEX2 replicates. 438 proteins were identified in both PsbU-APEX2 and both GFP-APEX2 replicates (Fig. 3c).

**Table 1:** Proteins enriched by PsbU-APEX2

Gene locus	Gene name	Identified in GFP-APEX2 samples? (a)	Enrichment value mean (b)	Functional description	PCC 6803 homolog (c)	Previous localization in PCC 6803 (d)
A1910	*petA*	None	11.2	Apocytochrome f precursor (cyt b6f)	sll1317	Thylakoid membrane (1, 2, 3, 4, 5, 6, 7), Inner membrane (5)
A1605	*psbQ*	None	10.88	PSII lumenal extrinsic	sll1638	Thylakoid membrane (2, 4, 5, 8, 9, 10), Inner membrane (5, 11, 12), Outer membrane (13)
A2294	*ppiB*	None	10.32	Putative peptidyl-prolyl cis–trans isomerase	sll0408	Thylakoid membrane (4, 5, 6, 10, 14, 15), Inner membrane (5)
A2695		None	10.18	Thylakoid-specific thioredoxin	slr1796	Thylakoid membrane (4, 10, 16)
A2730	*rfrC*	None	10.06	Manganese uptake-type porter	sll0274	Thylakoid membrane (4, 6, 15), Extracellular (17)
A0783		None	8.82	RfrA-like protein	sll0301	Thylakoid membrane (15)
A2577	*pqqE*	None	8.81	Peptidase, insulinase	sll0915	Thylakoid membrane (4, 6), Inner membrane (5), Outer membrane (13, 15), Periplasm (15, 18, 19), Extracellular (17)
A0477	*ymxG*	None	8.54	Zn-dependent peptidase	slr1331	Thylakoid membrane (6), Periplasm (15, 18), Extracellular (17)
A0322	*psbU*	One	8.22	PSII lumenal extrinsic	sll1194	Thylakoid membrane (1, 4, 5, 6, 8, 15, 22), Inner membrane (5), Extracellular (17)
A2619	*psb32*	None	8.07	PSII repair	sll1390	Thylakoid membrane (2, 4, 5, 6, 8, 15, 20), Inner membrane (20)
A0101		None	7.64	ICE-like protease (Caspase) p20 domain protein	sll0148	Thylakoid membrane (4, 6)
A1952		None	7.64	Endonuclease/ exonuclease/phosphatase or phytase	slr1087	Thylakoid membrane (4)
A0229	*ycf48*	None	7.58	PSII assembly	slr2034	Thylakoid membrane (1, 2, 4, 5, 14, 15, 21), Inner membrane (5), PratA-defined membrane (21)
A1522		None	7.52	Biotin carboxylase	slr1273	Thylakoid membrane (4)
A0604	*ycf51*	None	7.48	Unknown	sll1702	Thylakoid membrane (4, 5)
A0167	*petJ*	None	7.43	Cytochrome c6	sll1796	Thylakoid membrane (6)
A0269	*psbO*	Both	7.31	PSII lumenal extrinsic	sll0427	Thylakoid membrane (1, 4, 5, 6, 8, 10, 22, 23, 24, 25), Inner membrane (5, 11, 12, 24, 26)
A0843	*ctpA*	None	7.24	PSII assembly; carboxy-terminal-processing protease	slr0008	Thylakoid membrane (4), Inner membrane (24)
A1303	*psbP*	One	7.23	PSII assembly	sll1418	Thylakoid membrane (2, 4, 5, 14, 15, 27)
A1899		None	7.09	TPR domain protein of unknown function	sll0886	Thylakoid membrane (4)
A2533	*psb27*	None	7.09	PSII assembly	slr1645	Thylakoid membrane (1, 2, 4, 5, 8, 14, 15), Inner membrane (5)
A0806		One	6.87	Putative metalloendopeptidase	slr1579	Thylakoid membrane (5)
A2166		None	6.68	Unknown	slr1260	Membrane (28, 29), Soluble (29)
A0275		None	6.64	CAAX amino terminal protease family membrane protein	slr0959	Thylakoid membrane (2)
A2573		None	6.57	Alpha-2-macroglobulin; protease inhibitor	no homolog	
A1950		None	6.51	Unknown	slr0695	Thylakoid membrane (3, 6), Inner membrane (5, 11, 15), Outer membrane (13, 15)
A0948		None	6.5	Unknown	sll0066	Thylakoid membrane (5)
A1092	*ppib*	None	6.45	Putative peptidyl-prolyl cis–trans isomerase	sll0227	Thylakoid membrane (4, 5, 6), Inner membrane (5, 12, 15), Periplasm (15, 18, 19)
A1973	*ndhD2*	None	6.41	NDH complex	no homolog	
A2547	*ndhB*	One	6.13	NDH complex	sll0223	Thylakoid membrane (2, 4, 6, 30)
A0109	*bcp*	None	6.07	ROS scavenging; peroxiredoxin	sll0221	Membrane (28, 31), Soluble (2, 28, 29)
A2165		None	6.03	Unknown	sll5034	Thylakoid membrane (4)
A0238		None	6.02	Purine nucleoside permease	no homolog	
A1909	*petC*	One	5.96	Cytochrome b6-f complex iron-sulfur subunit	sll1316	Thylakoid membrane (2, 4, 5, 6, 7, 10, 22, 32), Inner membrane (5)
A0112	*psbV*	One	5.95	PSII lumenal extrinsic	sll0258	Thylakoid membrane (1, 4, 5, 6, 8, 9, 15), Inner membrane (5), Extracellular (17)
A0086		None	5.94	Oxidoreductase with molybdopterin binding domain, oxidizes thioredoxin	no homolog	
A0775		None	5.94	Histidinol phosphatase	no homolog	
A0189		None	5.93	Periplasmic Component of the Tol biopolymer transport system	slr1721	Inner membrane (2, 4, 5, 33)
A2605		None	5.93	S-layer protein	slr1704	Inner membrane (4, 5), Outer membrane (15), Extracellular (17)
A1987	*ytfC*	Both	5.9	FKBP-type peptidyl-prolyl cis–trans isomerase	slr1761	Thylakoid membrane (4, 6, 14), Periplasm (15, 18), Extracellular (17)
A1248	*crtU*	None	5.88	Beta-carotene desaturase	sll0254	Thylakoid membrane (2, 6)
A0190		None	5.88	Periplasmic component of the Tol biopolymer transport system	sll0325	Inner membrane (2, 4, 12, 15)
A1431		None	5.86	Unknown	slr1173	Thylakoid membrane (4)
A2116		None	5.8	Tfp pilus assembly protein FimV	no homolog	
A1008	*psaF*	One	5.71	PSI integral	sll0819	Thylakoid membrane (1, 2, 3, 4, 5, 6, 10, 24), Inner membrane (5, 11, 12, 34), Extracellular (17)
A1961	*psaA*	Both	5.62	PSI core	slr1834	Thylakoid membrane (1, 2, 3, 4, 5, 6, 21, 24), Inner membrane (5, 24), Extracellular (17)
A0728		None	5.61	Na^+^-dependent transporters of the SNF family	sll1486	Inner membrane (2, 4)
A2202	*rfrO*	None	5.58	Metal porter; pentapeptide repeat protein	sll0577	Membrane (28, 31), Soluble (28)
A2372	*napA*	None	5.57	Thylakoid sodium-proton antiporter	no homolog	
A0425	*pratA*	None	5.51	PSII assembly	slr2048	Inner membrane (35), PratA-defined membrane (21), Periplasm (15, 18, 36)
A1962	*psaB*	Both	5.49	PSI core	slr1835	Thylakoid membrane (1, 2, 3, 5, 6, 10, 24), Inner membrane (5, 24), Extracellular (17)
A0776		None	5.46	Histidinol phosphatase	no homolog	
A0842	*petB*	Both	5.42	Cytochrome b6	slr0342	Thylakoid membrane (2, 3, 5, 6, 7, 10)
A1093	*ycf4*	One	5.27	PSI assembly factor	sll0226	Thylakoid membrane (2, 4, 5), Inner membrane (5, 24)
A1433		None	5.18	Unknown	slr0431	Thylakoid membrane (22), Inner membrane (11, 15), Outer membrane (13, 15)
A2606		One	5.07	Prohibitin	slr1106	Thylakoid membrane (2, 4, 5, 6, 37), Inner membrane (11, 12, 26, 37)
A0339	*pbpB*	None	4.95	Penicillin-binding protein 2; important for heterocyst differentiation	no homolog	
A1956		None	4.83	Cofactor assembly of complex C subunit B	slr0589	Membrane (28, 29)
A0578	*amiC*	None	4.83	N-acetylmuramoyl-L-alanine amidase	slr1744	Inner membrane (2, 4, 5), Periplasm (15, 18), Extracellular (17)
A1330		Both	4.7	Glycine-rich membrane protein	slr0404	Thylakoid membrane (5), Inner membrane (5)
A1557		None	4.7	FHA domain protein	slr1624	Thylakoid membrane (2, 4, 5, 6), Inner membrane (5, 11, 15, 26), Extracellular (17)
A0737	*atpG*	None	4.69	ATP synthase	sll1323	Thylakoid membrane (2, 3, 4, 5, 6, 10), Inner membrane (5, 11, 12)
A0213	*prc*	None	4.68	Carboxyl-terminal protease	slr1751	Thylakoid membrane (6, 10), Inner membrane (5, 11), Outer membrane (13), Periplasm (18, 19), Extracellular (17, 38, 39)
A1244	*trxA*	None	4.66	Thioredoxin-like protein; txlA-like protein	sll1980	Thylakoid membrane (2, 5, 10, 15), Inner membrane (5)
A1127		None	4.63	Unknown	no homolog	
A0854	*ndhF*	Both	4.55	NDH complex	slr0844	Thylakoid membrane (2, 4)
A2620	*psaL*	Both	4.45	PSI integral	slr1655	Thylakoid membrane (1, 2, 3, 4, 5, 24), Inner membrane (5), Extracellular (17)
A1363	*manS*	None	4.43	Two-component sensor histidine kinase	slr0640	Membrane (28, 29, 31)
A0656	*secD*	Both	4.41	Protein translocase subunit	slr0774	Thylakoid membrane (5)
A0064	*ushA*	None	4.39	Bifunctional metallophosphatase/5′-nucleotidase	slr0306	Inner membrane (4), Outer membrane (15)
A2019	*oppA*	None	4.38	ABC-type dipeptide transport system, periplasmic component	sll1699	Inner membrane (2, 4, 5, 11, 12, 15, 34)
A1574		None	4.36	Efflux transporter	sll0180	Thylakoid membrane (5), Inner membrane (2, 5, 11, 12, 15, 26, 33, 34), Outer membrane (13, 15)
A1231		None	4.34	OmpA/MotB outer membrane porin	no homolog	
A0794	*sac1*	None	4.3	Sodium/sulfate symporter	sll0640	Inner membrane (2, 4)
A1405	*nblS*	Both	4.17	2-component system sensor for light stress/nutrient stress	sll0698	Thylakoid membrane (2, 4, 5, 6)
A1088	*ccs1*	One	4.1	Cytochrome c biogenesis protein CcsB	slr2087	Thylakoid membrane (4)
A0504	*gspD*	None	4.09	General secretion pathway protein D	slr1277	Thylakoid membrane (22), Inner membrane (4, 26), Outer membrane (13, 15)
A0465		None	4.03	Bestrophin family anion channel-forming protein	sll1024	Membrane (29)
A0398	*livK*	One	3.98	Periplasmic-binding protein of a branched-chain amino acid ABC transporter	slr0447	Thylakoid membrane (5, 6), Inner membrane (2, 4, 5, 11, 12, 15, 33, 34), Outer membrane (15), Periplasm (15, 18), Extracellular (17, 39)
A0230	*psbE*	None	3.96	PSII integral	ssr3451	Thylakoid membrane (1, 2, 3, 4, 5, 8, 24), Inner membrane (24)
A0381		One	3.94	Methanol dehydrogenase	sll1071	Thylakoid membrane (2, 15)
A0727	*ctaCII*	One	3.93	Cytochrome C oxidase	sll0813	Inner membrane (2, 4, 11, 12, 15, 33, 34)
A1897		None	3.92	Unknown	slr1177	Inner membrane (2)
A0445		None	3.92	Extracellular solute binding protein specific for oligopeptides	slr1740	Thylakoid membrane (5), Inner membrane (2, 4, 5, 12, 15, 26)
A2847		None	3.88	Unknown	no homolog	
A2578		One	3.82	Unknown	no homolog	
A1589	*psaC*	Both	3.79	PSI	ssl0563	Thylakoid membrane (1, 4, 5, 6, 10, 24), Inner membrane (5, 11, 24), Extracellular (17)
A0767	*lepB*	None	3.77	Signal peptidase I	sll0716	Thylakoid membrane (4, 10), Inner membrane (2)
A1638		None	3.68	Cell wall hydrolase, SpoIID-like protein	slr0191	Thylakoid membrane (6), Periplasm (15, 18)
A0349	*ftsH2*	Both	3.67	PSII repair	slr0228	Thylakoid membrane (2, 4, 5, 8, 15, 40, 41), Inner membrane (5)
A2000	*ndhD1*	None	3.63	NDH complex	slr0331	Thylakoid membrane (2, 6)
A1654	*hhoA/degQ*	None	3.55	Serine protease	sll1679	Thylakoid membrane (42), Inner membrane (2, 5, 12, 15, 42), Periplasm (15, 18)
G0157		None	3.52	Unknown	no homolog	
A2556		None	3.52	Peptidase	sll1488	No localization data
A1020		None	3.5	S-layer protein	slr1431	Inner membrane (2)
A0736	*atpF*	One	3.45	ATP synthase	sll1324	Thylakoid membrane (1, 2, 3, 4, 5, 6, 10), Inner membrane (5, 11, 12)
A1076		None	3.43	Unknown	slr0971	Inner membrane (2, 4)
A1207		None	3.38	Unknown	no homolog	
A2507	*sufA*	None	3.3	ABC-type Fe^3+^ transport system, periplasmic component (A1 iron uptake protein)	slr1295	Thylakoid membrane (5, 6, 10), Inner membrane (2, 5, 11, 12, 26, 33, 34), Outer membrane (13), Extracellular (17)
A2551		None	3.25	Double zinc ribbon and ankyrin repeat-containing protein	sll0283	Inner membrane (2, 4)
A1379	*spr*	None	3.18	Subtilisin-like serine protease	slr0535	Inner membrane (2, 4, 5)
A1964		None	3.15	Permease	no homolog	
A0682	*psaD*	Both	3.1	PSI	slr0737	Thylakoid membrane (1, 2, 3, 4, 5, 6, 10, 22, 24, 25), Inner membrane (5, 11, 24)
A1844	*ctpB*	None	3.02	Periplasmic carboxyl-terminal processing serine protease	slr0257	Periplasm (15, 18, 19)
A2435	*csgG*	None	2.96	Curli production assembly/transport component	sll1835	Thylakoid membrane (6, 22), Inner membrane (4, 11, 12, 15, 26), Outer membrane (13, 15), Periplasm (15, 18, 19), Extracellular (17, 39)
A2439		None	2.78	Unknown	no homolog	
A2176	*ycf37*	None	2.72	PSI assembly; Tetratricopeptide repeat-containing protein	slr0171	Thylakoid membrane (2, 4, 5, 21)
A1600	*Rho*	None	2.69	Unknown	slr0226	Inner membrane (2)
A1371	*mlaE*	None	2.69	ABC-type phospholipid uptake system permease component	slr1045	Inner membrane (4)
A2400		One	2.65	Thylakoid membrane protein of unknown function	slr0575	Thylakoid membrane (2, 4, 5, 6, 10, 15)
A1418; A0157; A2164	*psbA; psbAII; psbA*	Both	2.65	PSII core	sll1867; slr1311	Thylakoid membrane (1, 2, 3, 5, 6, 8, 10, 21, 24), Inner membrane (5, 24), PratA-defined membrane (21)
A1047	*secY*	Both	2.64	Protein translocase subunit	sll1814	Thylakoid membrane (2)
A1759	*psbB*	Both	2.63	PSII core	slr0906	Thylakoid membrane (1, 2, 3, 5, 7, 8, 21, 24, 37), Inner membrane (5)
A0991	*yidC*	Both	2.58	Membrane protein insertase YidC	slr1471	Thylakoid membrane (1, 2, 4, 5, 21, 35), Inner membrane (35), PratA-defined membrane (21)
A2056		One	2.56	Serine hydrolase	sll0854	Membrane (28, 29), Soluble (2, 28, 29, 43)
A1559	*psbC*	Both	2.47	PSII core	sll0851	Thylakoid membrane (1, 2, 3, 4, 5, 6, 8, 16, 20, 21, 24, 25, 30, 42, 44, 45), Inner membrane (5)
A2441		None	2.33	TetR family transcriptional regulator	sll1392	No localization data
A2554		None	2.29	Sugar ABC transporter ATP-binding protein	slr1224	Inner membrane (2, 4, 5)
A1664		None	2.17	Unknown	sll1573	Soluble (2, 29)
A2324	*bvdR*	None	2.09	Biliverdin reductase	slr1784	Membrane (28, 29, 31), Soluble (28, 29, 43)
A0926	*ndhA*	Both	2.02	NDH complex	sll0519	Thylakoid membrane (1, 2, 4, 5, 6)
A0091	*thrB*	None	2	Homoserine kinase	sll1760	Membrane (28, 29), Soluble (2, 28, 29)
G0011		One	1.95	Conserved Outer Membrane Protein	slr0042	Inner membrane (4), Extracellular (39)

(a) Number of GFP-APEX2 samples (2 total) that identified this protein. All proteins in this list were identified in both PsbU-APEX2 samples. (b) The mean enrichment value of the protein from all 4 analyses, higher enrichment values represent greater enrichment in PsbU-APEX2 samples over GFP-APEX2 samples. (c) Homologs are best reciprocal BLAST hit protein pairs between PCC 7002 and PCC 6803. (d) Localizations of soluble or membrane were only included if a more specific localization was not found. 1: Agarwal et al. (2010). 2: Baers et al. (2019). 3: Herranen et al. (2004). 4: Liberton et al. (2016). 5: Pisareva et al. (2011). 6: Rowland et al. (2010). 7: Schultze et al. (2009). 8: Kashino et al. (2002). 9: Kashino et al. (2006). 10: Srivastava et al. (2005). 11: Huang et al. (2002). 12: Huang et al. (2006). 13: Huang et al. (2004). 14: Heinz et al. (2016). 15: Rajalahti et al. (2007). 16: Zhu et al. (2016) 17: Gao et al. (2014a). 18: Fulda et al. (2000). 19: Kurian et al. (2006a). 20: Wegener et al. (2011) 21: Rengstl et al. (2011). 22: Wang et al. (2000). 23: Fulda et al. (2002). 24: Zak et al. (2001). 25: Zak et al. (1999). 26: Li et al. (2012). 27: Ishikawa et al. (2005). 28: Gao et al. (2014b). 29: Gao et al. (2015). 30: Ohkawa et al. (2002). 31: Zhang et al. (2013). 32: Aldridge et al. (2008). 33: Pisareva et al. (2007). 34: Zhang et al. (2009). 35: Selão et al. (2016). 36: Klinkert et al. (2004). 37: Boehm et al. (2009). 38: Cheregi et al. (2015). 39: Oliveira et al. (2016). 40: Komenda et al. (2006). 41: Sacharz et al. (2015). 42: Roberts et al. (2012) 43: Gan et al. (2005). 44: Xu et al. (2008). 45: Zhang et al. (2004).

### Biotinylated proteins enriched in PsbU-APEX2 samples

Mass spectrometry data were further analyzed to determine which proteins were labeled by PsbU-APEX2. PsbU is a lumenal extrinsic subunit of PSII and therefore the majority of PsbU-APEX2 is expected to be localized to the thylakoid membrane or lumen. However, because PsbU-APEX2 is translated in the cytoplasm and then translocated to its final destination in the lumen, we also expected that a small population of PsbU-APEX2 could be present in the cytoplasm, resulting in labeling of cytoplasmic proteins. Therefore, GFP-APEX2 was used as a control instead of a sample lacking APEX2/BP/H_2_O_2_, since it would control for the small cytoplasmic population of PsbU-APEX2 in addition to proteins nonspecifically bound to the streptavidin beads and endogenously biotinylated proteins.

An analysis of the mass spectrometry data using MaxQuant Label Free Quantitation (LFQ) intensities and normalized spectral counts was used to determine the identity of proteins specifically enriched with PsbU-APEX2 compared to the GFP-APEX2 control (Old et al. 2005). As part of this analysis, proteins were organized by descending enrichment value (log_2_(PsbU-APEX2 LFQ intensity/GFP-APEX2 LFQ intensity) or log_2_(PsbU-APEX2 normalized spectral counts/GFP-APEX2 normalized spectral counts). A true-positive list was constructed from PCC 7002 proteins homologous to *Synechocystis* sp. PCC 6803 (PCC 6803) proteins with evidence for thylakoid lumen or thylakoid membrane localization (Agarwal et al. 2010; Aldridge et al. 2008; Baers et al. 2019; Fulda et al. 2002; Heinz et al. 2016; Herranen et al. 2004; Kashino et al. 2002, 2006; Komenda et al. 2006; Liberton et al. 2016; Ohkawa et al. 2002; Pisareva et al. 2011; Rajalahti et al. 2007; Rengstl et al. 2011; Rowland et al. 2010; Sacharz et al. 2015; Schultze et al. 2009; Srivastava et al. 2005; Wang et al. 2000; Xu et al. 2008; Zak et al. 1999, 2001; Zhang et al. 2004). A false-positive list of PCC 7002 proteins was constructed from homologous proteins found in the soluble proteome of PCC 6803 that do not have signal sequences or transmembrane helices, as these proteins are expected to be cytoplasmic (Baers et al. 2019; Choi et al. 2000; Fulda et al. 2006; Fuszard et al. 2013; Gan et al. 2005; Gao et al. 2014b, 2015, 2009; Kurian et al. 2006b; Mata-Cabana et al. 2007; Mehta et al. 2014; Mikkat et al. 2014; Pandhal et al. 2009; Pérez‐Pérez et al. 2006; Plohnke et al. 2015; Rowland et al. 2011; Simon et al. 2002; Slabas et al. 2006). As expected, proteins from the true-positive list have significantly higher enrichment values than proteins from the false-positive list (Fig. S2). Using the true- and false-positive lists, we identified a cutoff value to discriminate between enriched proteins and those that bound to the beads non-specifically or were enriched by GFP-APEX2. This analysis was performed using both enrichment values for both PsbU-APEX2 replicates (Table S1). Therefore, two analyses were performed on each PsbU-APEX2 replicate, one using enrichment values calculated with LFQ intensity values and a second using enrichment values calculated with normalized spectral counts. To be as stringent as possible, only the 123 proteins above the cutoff in all four analyses were reported, which we called PsbU-APEX2-enriched proteins (Table 1). The PsbU-APEX2 enriched proteins include a subset of the 99 proteins exclusive to the PsbU-APEX2 replicates, as well as additional proteins enriched in abundance over the GFP-APEX2 replicates. Major functions of enriched proteins are shown in Fig. 3d.

The list of 123 PsbU-APEX2 enriched proteins includes many proteins expected to be present within the thylakoid lumen and membrane (Table 1). The majority of proteins (73) have PCC 6803 homologs previously localized to thylakoid membrane or lumen (See Table 1). Out of the 50 proteins that have not been previously localized to the thylakoid membrane, 17 have no PCC 6803 homolog, 12 have no localization data for specific cellular structures or regions, and 21 have only previously been localized to somewhere other than the thylakoid membrane or lumen, such as the plasma membrane or periplasm. This analysis of previous localizations of homologous proteins in the literature was performed in lieu of experimental validation of the localization of enriched proteins. There is no other method to biochemically separate the thylakoid lumen from other intracellular structures, and while fluorescence microscopy of GFP-tagged proteins could be used to determine if a protein associates with the thylakoid membranes, it does not have the resolution to determine if a protein is on the cytoplasmic or lumenal side of the thylakoid membrane. Previous localizations of homologous proteins were used because most localization studies in cyanobacteria have been done in other species, specifically PCC 6803, and very few have been completed in PCC 7002. To further support the hypothesis that PsbU-APEX2 enriched proteins are part of a cellular compartment and not cytoplasmic, the presence of signal sequences and transmembrane helices were predicted from their protein sequences (see Table S2). The majority (105) of enriched proteins possess either a signal sequence or at least one transmembrane helix.

**Fig. 4:**
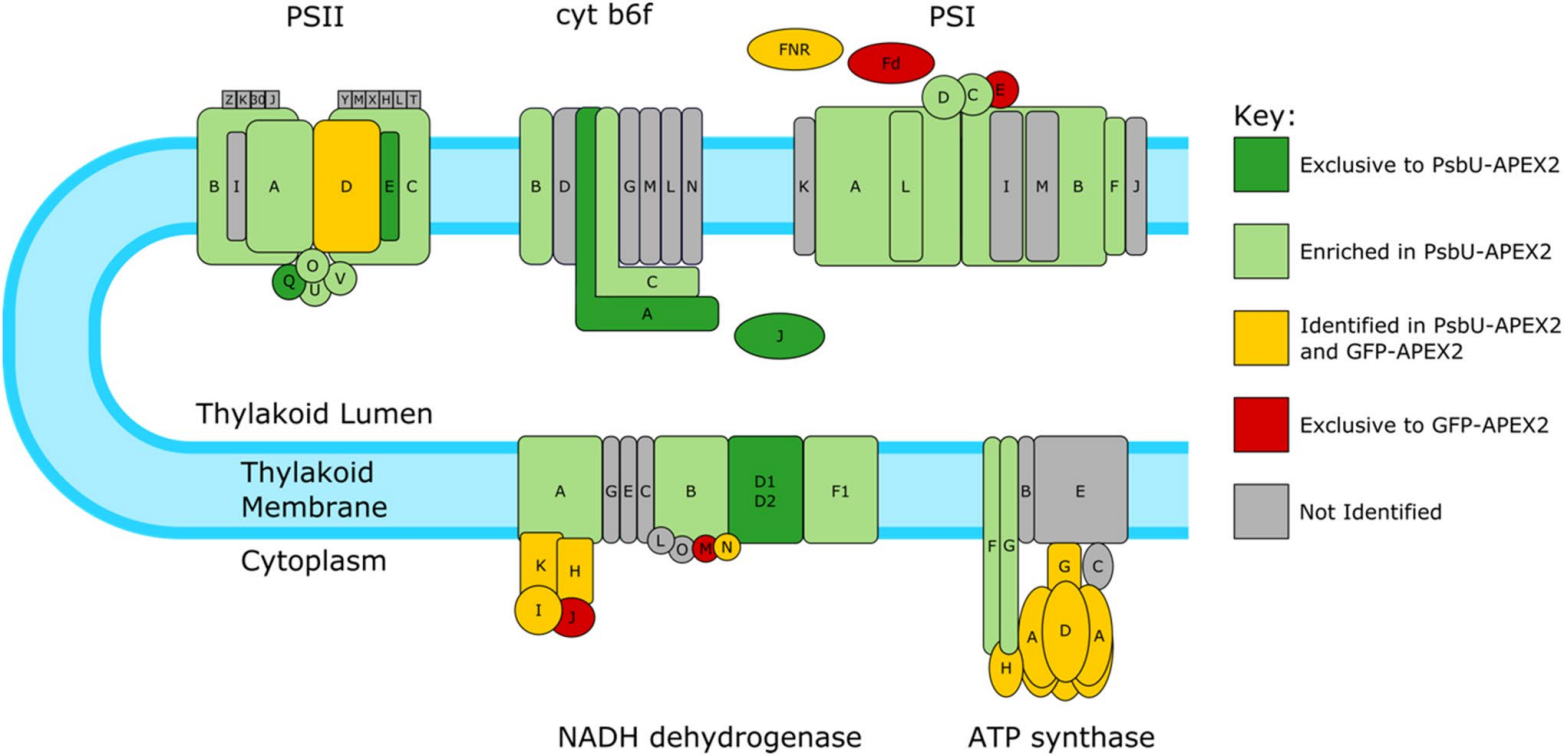
Enrichment of Protein Complex Subunits in the Thylakoid Membrane. The protein complexes present in the thylakoid membrane are color-coded by their enrichment; the key is located on the right side of the figure. Light and dark green subunits are both enriched in the PsbU-APEX2 samples over the GFP-APEX2 samples; the dark green samples were unique to the PsbU-APEX2 samples, while the light green subunits were also identified in the GFP-APEX2. Yellow subunits represent proteins identified in both PsbU-APEX2 and GFP-APEX2 samples but not enriched in PsbU-APEX2. Red subunits are proteins unique to the GFP-APEX2 samples. Gray proteins were not identified by mass spectrometry in this study. The identity of each protein complexes is either above or below the complex. The proteins associated with a specific complex are named with the following prefixes followed by the letter or number the protein is labeled with: Psb for PSII, Pet for cyt b_6_f, Psa for PSI, Ndh for NADH dehydrogenase, and Atp for ATP synthase. The exceptions to this are Fd (PetF) and FNR (PetH). Note—there are two different proteins both called AtpG; the yellow subunit refers to A0733 and the light green subunit refers to A0737

Thylakoid lumen proteins, including the lumenal extrinsic subunits of PSII (PsbU, PsbQ, PsbO, and PsbV) and Cyt *c*_6_ (PetJ1) were enriched in PsbU-APEX2 samples (Fig. 4). Unlike PCC 6803, PCC 7002 does not express plastocyanin, and therefore, PetJ1 is the only protein known that is soluble in the thylakoid lumen and not tightly associated with a protein complex. This protein was enriched in our analysis, demonstrating that the technique used is able to enrich for soluble proteins within the thylakoid lumen. Additionally, enrichment of the PSII integral membrane subunits and extrinsic lumenal subunits shows the capability of APEX2 to label membrane-associated and integral membrane proteins. Integral membrane proteins from PSII, photosystem I (PSI), cytochrome b_6_f, ATP synthase, and NADH dehydrogenase (NDH), if identified by mass spectrometry, were enriched in the PsbU-APEX2 samples, with the exception of the PsbD subunit of PSII and the NdhD3 (A0173) and NdhF3 (A0172) subunits of NDH. PsbD and NdhF3 protein were above the enrichment cutoff in two analyses, but the below the cutoff in the other two analyses (Tables S1 and S2). The NdhD3 protein was above the cutoff in only one analysis. Most of the cytoplasmic non-membrane integral protein subunits of PSI, ATP synthase, and NDH complexes are not enriched in the PsbU-APEX2 samples, and some are unique to the GFP-APEX2 samples. The lack of enrichment of proteins on the cytoplasmic side of the thylakoid membrane demonstrates the specificity of PsbU-APEX2 to label proteins within the lumen and thylakoid membrane. The cytoplasmic facing subunits that were enriched in the PsbU-APEX2 samples are PsaC and PsaD. These subunits and PsaE are within the top 15% of proteins ranked by membrane association, and are more tightly associated with the membrane than the phycobilisome proteins and the cytoplasmic subunits of NDH and ATP synthase (Gao et al. 2015). PsbU-APEX2 will be more efficient at labeling cytoplasmic side proteins closely associated with the thylakoid membrane, like PsaC and PsaD, since proteins closely associated with the thylakoid membrane are within the biotinylation radius of lumenal PsbU-APEX2 for more time. Following that same logic, freely diffusing cytoplasmic GFP-APEX2 is likely more efficient at biotinylating freely diffusing cytoplasmic proteins than proteins closely associated with the thylakoid membrane.

**Fig. 5:**
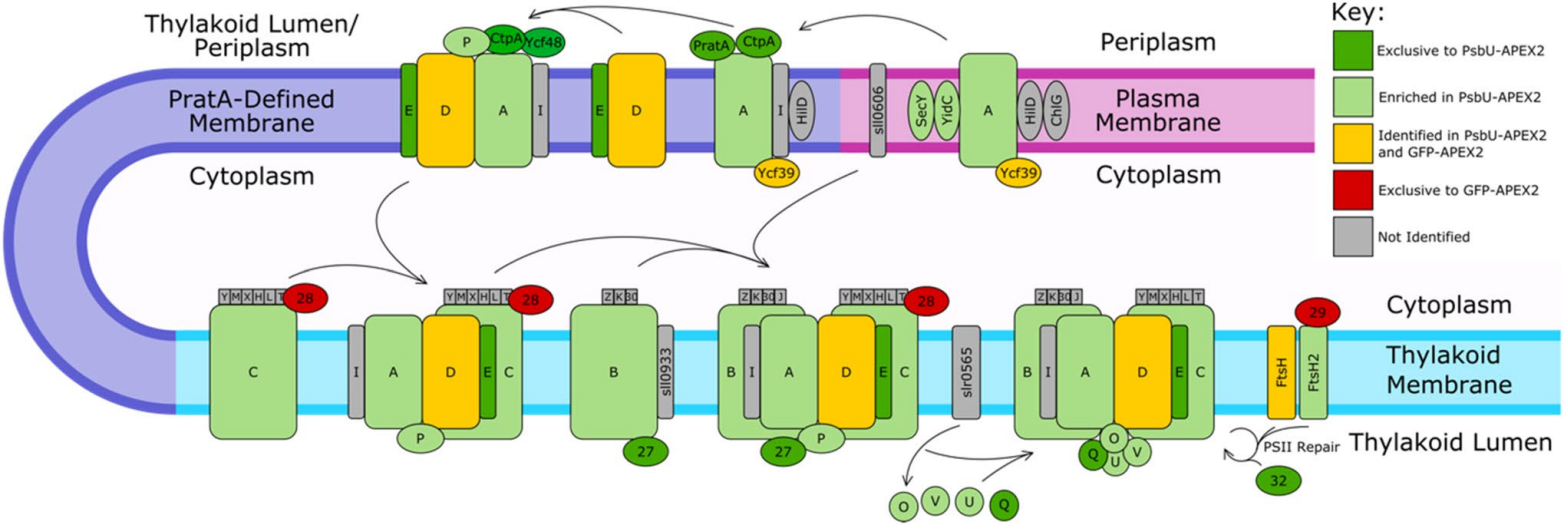
Enrichment of PSII assembly factors. The PSII assembly and repair components known in PCC 6803 are shown. The proteins are color-coded by their enrichment, the key is located on the top. Light and dark green subunits are both enriched in the PsbU-APEX2 samples over the GFP-APEX2 samples; the dark green samples were unique to the PsbU-APEX2 samples, while the light green subunits were also identified in the GFP-APEX2. Yellow subunits represent proteins identified in both PsbU-APEX2 and GFP-APEX2 samples but not enriched in PsbU-APEX2. Red subunits are proteins unique to the GFP-APEX2 samples. Gray proteins were not identified by mass spectrometry in this study. The prefix “Psb” should be added to any proteins labeled with only a letter or number to obtain the name of the protein

Many factors involved in the assembly of PSII were also PsbU-APEX2 enriched (Fig. 5). Proteins both early and late in the assembly process were enriched. SecY and Alb3, proteins involved in inserting the PsbA into the membrane were enriched (Chidgey et al. 2014; Linhartová et al. 2014; Sachelaru et al. 2013). PratA, a protein that is thought to deliver Mn^2+^ to PsbA, and CtpA, which processes the C-terminal tail of PsbA, have previously been localized to the periplasm and plasma membrane, respectively, but were exclusively found in PsbU-APEX2 samples in this study (Anbudurai et al. 1994; Klinkert et al. 2004; Komenda et al. 2006; Schottkowski et al. 2009; Stengel et al. 2012; Zak et al. 2001). PsbP, Ycf48, and Psb27 are PSII assembly factors enriched by PsbU-APEX2 that are thought to be localized within the thylakoid lumen (Heinz 2016). The assembly factors Ycf39 and Psb28, along with the PSII repair factor Psb29, are on the cytoplasmic side of membranes and were not enriched by PsbU-APEX2 (Bec̆ková et al. 2017; Heinz 2016). The lumenal proteins YtfC and A2294 (homologous to sll0408 in PCC 6803) are homologs to factors important for PSII assembly in plants that were also PsbU-APEX2 enriched (Heinz 2016). Proteins involved in PSII repair were also enriched by PsbU-APEX2. For example, Psb32, a protein that protects PSII from photodamage and aids in PSII repair, was exclusive to PsbU-APEX2 samples (Wegener et al. 2011). Additionally, FtsH2, a protein involved in the repair of damaged PSII, was also enriched in PsbU-APEX2 samples (Komenda et al. 2006, 2010). PsbQ, a protein present in the most active PSII fraction that is thought to define the complete assembly of PSII was also exclusive to PsbU-APEX2 (Roose et al. 2007). The variety of early and late assembly factors enriched by PsbU-APEX2 demonstrate the ability of APEX2-based proximity-based proteomics to capture assembly intermediates of protein complexes of low abundance. In the future, this technique could be used to gain novel insights into low abundance assembly intermediates of protein complexes in other processes.

Many proteins involved in other cellular processes were localized to the thylakoid membrane and lumen in this study. At least ten proteases were enriched in PsbU-APEX2, including the thylakoid signal peptidase LepB (Zhbanko et al. 2005). PsbU-APEX2 enriched proteins also include proteins involved in transport of numerous different known and unknown substrates. Many of the proteins involved in transport and protein trafficking, assembly, and processing have previously been localized to the periplasm or the plasma membrane, and have not been localized to the thylakoid membrane. Furthermore, many other proteins enriched by PsbU-APEX2 have been localized to the plasma membrane and/or the periplasm in addition to the thylakoid membrane. The biological relevance of the plasma membrane and periplasmic proteins enriched by PsbU-APEX2 is unclear. It is possibly an artifact of overexpression of PsbU-APEX2. However, the cyanobacterium *Gloeobacter violaceus* does not contain a thylakoid membrane (Mareš et al. 2013) and instead performs oxygenic photosynthesis in the inner membrane. If the thylakoid membrane and lumen originated from the plasma membrane and periplasmic space, respectively, perhaps it is not surprising that some proteins are found in both cellular fractions. Furthermore, ultrastructural studies of PCC 6803 using cryo-electron tomography identified sites of contact between the thylakoid and plasma membrane (Rast et al. 2019). Additional possibilities include dual localization of proteins, low fidelity of the sorting mechanism of translocated proteins into the lumen and the periplasm, and post-translocation sorting of proteins into their final localization. Further experiments are needed to determine the biological relevance of the periplasmic and inner membrane proteins observed.

In addition to large protein complexes involved in energy metabolism, PSII assembly factors, and proteases, the PsbU-APEX2-enriched proteins include proteins with other functions. For example, several thioredoxins, including the thylakoid specific thioredoxin A2695, were enriched (Zhu et al. 2016). A beta-carotene desaturase (A1248) was also identified. Proteins involved in maintaining the cell wall (A0339 and A0578) and S-layer proteins (A2605 and A1020) were also enriched. Another protein (A1522) with homology to biotin carboxylases was also enriched by PsbU-APEX2 in this study. Additionally, there are several proteins that have not been previously localized and have unknown functions (A1127, A1207, A1664, A2166, A2439, A2578, A2847, and G0157). These proteins could be the subject of future research.

The experiments performed here demonstrate the potential of APEX2 to interrogate the proteome of regions of cyanobacteria that have not been previously biochemically purified, like the thylakoid lumen. It also demonstrates the ability of APEX2 to capture low abundance protein complex assembly intermediates. In the future, this technique can be used to monitor the proteomes of specific regions of the cell under different environmental conditions. Additionally, APEX2 can be used to determine the topology of membrane proteins and identify candidates for protein–protein interactions (Lee et al. 2016; Lobingier et al. 2017; Mavylutov et al. 2018; Paek et al. 2017). Proximity-based proteomics using APEX2 has the potential to be a powerful tool in the pursuit of understanding the physiology of photosynthetic organisms.

## Methods

### Creation of PCC 7002 strains

The *psbU* gene (SynPCC7002_A0322) was amplified from PCC 7002 while APEX2 was amplified from a plasmid gifted to us by Alice Ting (Addgene plasmid # 72,558; http://n2t.net/addgene:72558; RRID:Addgene_72558). Plasmids were assembled using Gibson Assembly (Gibson et al. 2009) with neutral site 1 as the homology arms, p_*ccmK2*_ as the promoter (Cameron et al. 2013; Ruffing et al. 2016), and kanamycin resistance for selection. The Gibson reactions were transformed into DH5α *E. coli*, and minipreps of liquid cultures started from single colonies were performed to collect plasmid. Plasmid was transformed into PCC 7002 (Stevens and Porter, 1980) and colonies containing the desired insert were serially passaged in the presence of antibiotic until segregated.

### Biotinylation of proteins by APEX2 in PCC 7002

Biotinylation of proteins was performed using a modified protocol from Hung et al. and Hwang and Espenshade that was optimized for PCC 7002 (2016; 2016). Briefly, 50 mL cultures of PCC 7002 strains were grown in A + media (Stevens et al. 1973) in air at 37 °C with a light intensity of 185 µmol photons m^−2^ s^−1^ for 2 days to an OD_730_ of about 0.5. Several µL of culture were saved to image on the microscope. The culture was pelleted at 4300×*g* for 10 min at 4 °C. The supernatant was poured off and cells were resuspended in 4 mL A + medium with 2.5 mM BP and transferred to a six-well plate. Six-well plates were incubated shaking in air at 37 °C with a light intensity of 185 µmol photons m^−2^ s^−1^ for 30 min. Samples were then pelleted in a 1.5 mL tube and resuspended in 1 mL phosphate buffered saline pH 7.8 (Bio-Rad) (PBS). 10 µL of 100 mM H_2_O_2_ was added and cells were inverted for 30 s before pelleting for 30 s. Supernatant was removed and cells were resuspended in quencher solution (PBS with 10 mM sodium ascorbate, 5 mM Trolox and 10 mM sodium azide) and pelleted. This step was repeated two additional times. The supernatant was removed and the cell pellets were frozen at − 80 °C for storage and to facilitate cell lysis.

### Cell lysis

The cell pellet was resuspended in RIPA lysis buffer with quenchers (50 mM Tris pH 7.4, 150 mM NaCl, 0.1% (w/v) SDS, 0.5% (w/v) sodium deoxycholate, 1% (v/v) Triton X-100, 10 mM sodium ascorbate, 5 mM Trolox, 10 mM sodium azide, 1 mM PMSF). Cells were lysed using bead beating, with 30 cycles of 20 s on and 20 s off on ice. The lysate and beads were pelleted at 2000×*g* and the supernatant was collected. The supernatant was then pelleted for 5 min at 15000×*g* and the supernatant was collected and flash frozen.

### Protein concentration measurement

The protein concentration of cell lysate was quantified using the Pierce 660 nm Protein Assay (Thermo Fisher).

### Purification of biotinylated proteins

Streptavidin magnetic beads (Pierce) were washed twice in RIPA lysis buffer (50 mM Tris pH 7.4, 150 mM NaCl, 0.1% (w/v) SDS, 0.5% (w/v) sodium deoxycholate, 1% (v/v) Triton X-100) and the supernatant was removed. 800 µL of RIPA lysis buffer with quenchers containing 50 µg of protein for every 50 µL of streptavidin magnetic beads was added. Beads were incubated with protein for 1 h at room temperature on a rotator. The beads were then washed twice with RIPA lysis buffer, once with 1 M KCl, once with 0.1 M Na_2_CO_3_, once with 8 M urea in 10 mM Tris pH 7.5, and once again with RIPA lysis buffer.

### Elution of biotinylated proteins for gels and blots

Beads were denatured at 98 °C for 10 min in 30 µL of elution buffer (3X Laemmli buffer, 2 mM biotin, 20 mM DTT) to elute biotinylated proteins. The eluate was collected and diluted with 60 µL of water to run on gels.

### Preparation for mass spectrometry

Beads were washed an additional 5 times with 50 mM NH_4_HCO_3_ containing 0.2% (w/v) sodium deoxycholate. The supernatant was removed and beads were resuspended in 50 µL 10 mM TCEP and 40 mM chloroacetamide and incubated at 37 °C for 30 min to reduce and alkylate the proteins. 150 µL water containing 0.225% (w/v) sodium deoxycholate and 0.2 µg Promega sequencing grade modified trypsin was added. An on-bead digestion was performed overnight on a rotator at 37 °C. Beads were pelleted and the supernatant was collected. Formic acid was added to 2% (w/v) to stop digestion. Sodium deoxycholate was removed using 3 phase transfers with ethyl acetate. The samples were desalted using in-house STAGE tips with 3 M Empore SDB-RPS membrane and dried using a vacuum centrifugation.

### LC–MS/MS

The tryptic peptides were resolved using an UltiMate 3000 UHPLC system (Thermo Fisher) in a direct injection mode. Peptides were reconstituted in Buffer A (0.1% formic acid in water), and peptide concentration was measured using Fluoraldehyde *o*-Phthaldialdehyde Reagent (Thermo Fisher). For each sample, 250 ng (5 µL) of the peptides were loaded onto a Waters BEH C18 column (130 Å, 1.7 µm × 75 µm × 250 mm) with 98.4% Buffer A and 1.6% Buffer B (0.1% formic acid in acetonitrile) at 0.4 µL/min for 16.67 min. Peptides were resolved and eluted using a gradient of 1.6 to 8% B (0–8 min), 8–20% B (8–140 min), and 20–32% B (140–160 min) at 0.3 µL/min. MS/MS was performed on a Q-Exactive HF-X mass spectrometer (Thermo Fisher), scanning precursor ions between 380–1580 m/z (60,000 resolution, 3 × 10^6^ ions AGC target, 45 ms maximum ion fill time), and selecting the 12 most intense ions for MS/MS (15,000 resolution, 1 × 10^5^ ions AGC target, 150 ms maximum ion fill time, 1.4 m/z isolation window, 27 NCE, 30 s dynamic exclusion). Ions with unassigned charge state, + 1, and >  + 7 were excluded from the MS/MS.

### Silver stain protocol

Proteins were separated on a 10% SDS-PAGE gel and stained using the short silver nitrate staining protocol described in by Chevallet et al. (2006).

### Immunoblotting

Proteins were separated on a 10% SDS-PAGE gel and immunoblots were performed following the protocol from Green and Sambrook (2012). Protein was transferred to a nitrocellulose membrane, or a polyvinylidene fluoride (PVDF) membrane if fluorescent secondary antibodies were used. After blocking membranes overnight, membranes were incubated with GFP (Invitrogen, cat. no. A6455) or RbcL (Agrisera, cat. no. AS03037) antibodies, or streptavidin-HRP (Life Technologies, cat. no. R960-25). Membranes probed for GFP or RbcL were then incubated with a secondary antibody conjugated to HRP or AlexaFluor 488 (Thermo Fisher, cat. no. A-11008 or cat. no. 31460). Membranes were visualized using chemiluminescence after exposure to the Clarity Western ECL substrate (Bio-Rad) or fluorescence. If necessary, blots were stripped using ReBlot Plus Mild Solution (Millipore).

### Fluorescence microscopy

Cells were spotted onto an agar pad (A + with 1% agar) and placed onto a microscope slide. Cells were imaged on a customized Nikon TiE inverted wide-field microscope with a Near-IR-based Perfect Focus system. Images were acquired with an ORCA Flash4.0 V2 + Digital sCMOS camera (Hamamatsu) using a Nikon CF160 Plan Apochromat Lambda 100 × oil immersion objective (1.45 N.A.). Chlorophyll fluorescence of thylakoid membranes was imaged using a 640 nm LED light source (SpectraX) for excitation and a standard Cy5 emission filter. GFP localization was imaged using a 470 nm LED light source (SpectraX) for excitation and a standard GFP emission filter.

### LC–MS/MS data analysis

MaxQuant/Andromeda (version 1.6.1.10) was used to process raw files from the Q Exactive HF-X and search the peak lists against a database consisting of Uniprot PCC 7002 proteome (UP000001688, total 3,179 entries, downloaded at 6/22/2019). The search allowed trypsin specificity with a maximum two missed-cleavage and set carbamidomethyl modification on cysteine as a fixed modification and protein N-terminal acetylation and oxidation on methionine as variable modifications. MaxQuant used 4.5 ppm main search tolerance for precursor ions, 20 ppm MS/MS match tolerance, searching top 12 peaks per 100 Da. False discovery rates for both protein and peptide were 0.01 with a minimum of seven amino acid peptide length. Label-free quantification was enabled with minimum 2 LFQ ratio counts and a fast LFQ option. The mass spectrometry proteomics data have been deposited to the ProteomeXchange Consortium via the PRIDE partner repository with the dataset identifier PXD021787 (Perez-Riverol et al. 2019).

Only proteins with at least two unique peptides and two spectral counts were considered identified in an individual sample. PCC 7002 proteins identified in both GFP-APEX2 replicates and/or both PsbU-APEX2 replicates were retained for further analysis, including the PsbU-APEX enriched protein analysis and the Venn diagram (Table S1). A presence/absence Venn diagram was constructed (Fig. 3c). A protein must be identified in both replicates of a sample to appear in the Venn diagram. Proteins identified in both replicates of a sample and only one replicate of the other sample (176 proteins) were not added to the Venn diagram as their localization was unclear.

### PsbU-APEX2 enriched protein analysis

The log_2_ ratio of the MAXQUANT LFQ intensities and the log_2_ ratio of normalized spectral counts were used as metrics to determine enrichment in the PsbU-APEX2 A and PsbU-APEX2 B samples over the GFP-APEX2 B sample (log_2_(U/G)) (Old et al. 2005). If a protein was not identified in a sample, the LFQ intensity was set to zero. To determine the cutoff for proteins enriched in PsbU-APEX2 samples, identified proteins were cross-referenced with true positive (TP) or false positive (FP) lists. The TP lists were assembled using localization data from studies of the thylakoid lumen or thylakoid membrane in PCC 6803. All proteins experimentally localized or predicted to localize to the thylakoid lumen in any study were included in the TP list (Aldridge et al. 2008; Fulda et al. 2002; Heinz et al. 2016; Kashino et al. 2006; Rajalahti et al. 2007). To include integral thylakoid membrane proteins, proteins localized to the thylakoid membrane in at least 4 studies that had at least 1 predicted transmembrane helix were also added to the TP list (Agarwal et al. 2010; Baers et al. 2019; Herranen et al. 2004; Kashino et al. 2002; Komenda et al. 2006; Liberton et al. 2016; Ohkawa et al. 2002; Pisareva et al. 2011; Rengstl et al. 2011; Rowland et al. 2010; Sacharz et al. 2015; Schultze et al. 2009; Srivastava et al. 2005; Wang et al. 2000; Xu et al. 2008; Zak et al. 1999, 2001; Zhang et al. 2004). The FP list was assembled using data from studies of the soluble proteome of PCC 6803. The FP list contained proteins that were found in the soluble proteome in at least 4 studies, had no predicted signal sequence or transmembrane helix, and was found in 1 or less studies of the thylakoid membrane (Baers et al. 2019; Choi et al. 2000; Fulda et al. 2006; Fuszard et al. 2013; Gan et al. 2005; Gao et al. 2014b, 2015, 2009; Kurian et al. 2006b; Mata-Cabana et al. 2007; Mehta et al. 2014; Mikkat et al. 2014; Pandhal et al. 2009; Pérez‐Pérez et al. 2006; Plohnke et al. 2015; Rowland et al. 2011; Simon et al. 2002; Slabas et al. 2006). The TP and FP lists are in Supplementary Table 3.

A total of four analyses were performed, one for each enrichment metric (Log_2_(U/G) using LFQ intensity and Log_2_(U/G) using normalized spectral counts) in each PsbU-APEX2 sample. For each protein in every analysis, the true positive rate (TPR) and the false-positive rate (FPR) were calculated. The TPR for a specific protein was the number of TP proteins with an enrichment greater than or equal to the enrichment of the specific protein divided by the total number of TP proteins found in the experiment. The FPR for a specific protein was the number of FP proteins with an enrichment greater than or equal to the enrichment of the specific protein divided by the total number of FP proteins. The cutoff for each sample was the enrichment with the greatest difference between the TPR and FPR value. The proteins above the cut-off of the in all 4 analyses are reported in Table 1 and were used to make Fig. 3d.

### Signal sequence prediction

To predict if a protein had a signal sequence and the cut site to the remove the signal sequence, all proteins in the UniProt reference proteome for PCC 7002 were analyzed with SignalP-5.0 using both the Gram-positive and Gram-negative bacterial options.

### Transmembrane helices prediction

To predict if a protein had transmembrane helices, all proteins in the UniProt reference proteome for PCC 7002 were analyzed using the TMHMM Server v. 2.0.

## Supplementary Information

Below is the link to the electronic supplementary material.

Supplementary file1 (XLSX 1320 KB)

Supplementary file2 (XLSX 78 KB)

Supplementary file3 (XLSX 14 KB)

## Data Availability

The authors confirm that the data supporting the findings of this study are available within the article and supplementary materials.

## Code availability

MaxQuant software was used to analyze mass spectrometry data. The software is available at https://www.maxquant.org/. SignalP 5.0 was used to predict signal sequences and is available at http://www.cbs.dtu.dk/services/SignalP/. Transmembrane helices were predicted using the TMHMM Server v 2.0 from http://www.cbs.dtu.dk/services/TMHMM/.
